# Suppressive Effects of a Truncated Inhibitor K562 Protein-Derived Peptide on Two Proinflammatory Cytokines, IL-17 and TNF-α

**DOI:** 10.4014/jmb.2004.04062

**Published:** 2020-09-22

**Authors:** Jong Tae Hwang, Ji Won Yu, Hee Jin Nam, Sun Kwang Song, Woo Yong Sung, Yongae Kim,, Jang-Hee Cho

**Affiliations:** 1 Biomaterial Research Center, Cellinbio, Suwon 668, Republic of Korea; 2 Department of Biology, Kongju National University, Kongju 3588, Republic of Korea; 3 Department of Chemistry, Hankuk University of Foreign Studies, Yongin 1705, Republic of Korea

**Keywords:** Anti-inflammatory, inhibitor K562, interleukin-17, truncated IK, tumor necrosis factor-α

## Abstract

Inhibitor K562 (IK) protein was first isolated from the culture medium of K562 cells, a leukemia cell line, and is an inhibitory regulator of interferon-γ-induced major histocompatibility complex class II expression. Recently, exogenous truncated IK (tIK) protein showed potential as a therapeutic agent for inflammation-related diseases. In this study, we designed a novel putative anti-inflammatory peptide derived from tIK protein based on homology modeling of the human interleukin-10 (hIL-10) structure, and investigated whether the peptide exerted inhibitory effects against proinflammatory cytokines such as IL-17 and tumor necrosis factor-α (TNF-α). The peptide contains key residues involved in binding hIL-10 to the IL-10 receptor, and exerted strong inhibitory effects on IL- 17 (43.8%) and TNF-α (50.7%). In addition, we used circular dichroism spectroscopy to confirm that the peptide is usually present in a random coil configuration in aqueous solution. In terms of toxicity, the peptide was found to be biologically safe. The mechanisms by which the short peptide derived from human tIK protein exerts inhibitory effects against IL-17 and TNF-α should be explored further. We also evaluated the feasibility of using this novel peptide in skincare products.

## Introduction

**Fig. 1:**
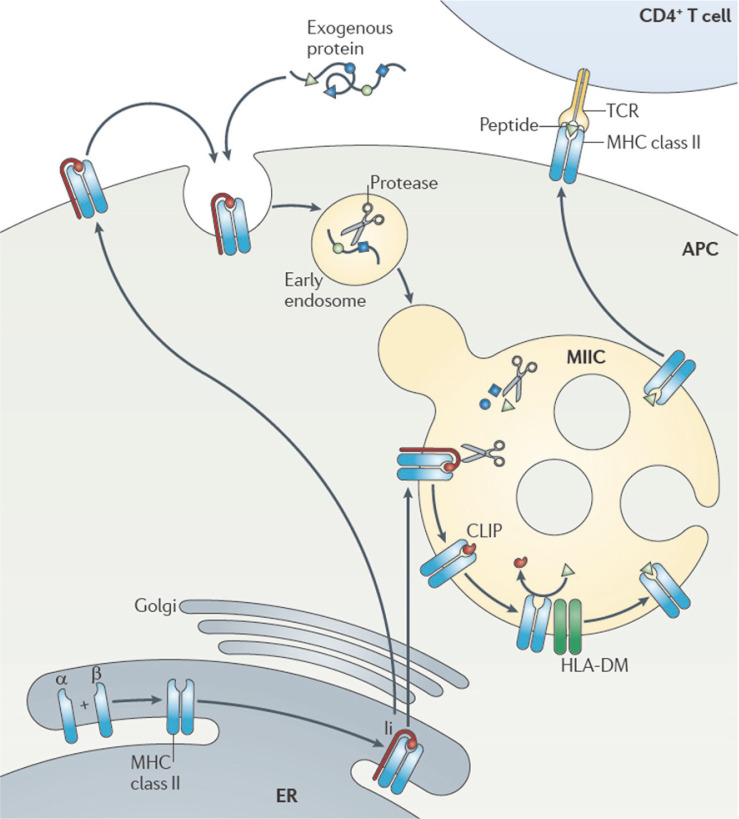
Schematic representation of the basic major histocompatibility complex (MHC) class II antigen-presentation pathway (data from Neefjes *et al*. [2]). ER, endoplasmic reticulum; li, invariant chain; MIIC, MHC class II compartment; CLIP, class II-associated Ii peptide; HLA-DM, an MHC-like molecule that acts as a chaperone in MHC class II peptide loading; APC, antigen-presenting cell; TCR, T-cell receptor. See text for a detailed description of the pathway.

Inhibitor K562 (IK) was first isolated from the culture medium of K562, a leukemia cell line. It downregulates interferon-γ-induced major histocompatibility complex (MHC) class II, which plays a key role in the immune response [1, 2]. MHC class II α- and β-chains assemble in the endoplasmic reticulum and form a complex with the invariant chain (Ii). The Ii-MHC class II heterotrimer is transported through the Golgi apparatus to the MHC class II compartment (MIIC), directly and/or via the plasma membrane. Endocytosed proteins and Ii are degraded by resident proteases in the MIIC. The class II-associated Ii peptide fragment of Ii remains in the peptide-binding groove of the MHC class II dimer, and is exchanged for an antigenic peptide with the help of a dedicated chaperone, human leukocyte antigen (HLA)-DM. MHC class II molecules are then transported to the plasma membrane to present antigenic peptides to CD4^+^ T cells (Fig. 1). Furthermore, the expression of MHC class II molecules can be induced in non-professional antigen-presenting cells (*e.g.*, fibroblasts, epithelial cells, and keratinocytes) in an environment rich in inflammatory cytokines, of which interferon-γ is the most potent [3-5]. In summary, MHC class II molecules can be expressed in immune cells, often in response to infections or inflammation. In addition, Cella *et al*. reported that the formation of peptide-MHC class II complexes is boosted by inflammatory stimuli that induce the maturation of dendritic cells [6].

The class II transactivator (CIITA) is necessary for both constitutive and interferon-γ-induced MHC class II expression. Examination of CIITA mRNA in clones stably transfected with IK revealed a marked reduction in CIITA mRNA transcription. Vedrenne *et al*. [7] suggested that the function of IK may be related to CIITA, which is influential in both constitutive and cytokine-inducible expression of MHC class II. Accordingly, excessive expression of IK negatively regulated CIITA mRNA transcription and reduced the expression level of MHC class II. Taken together, their results indicate that the IK protein plays a key role in the constitutive expression of MHC class II antigens, and that the inhibition induced by IK occurs upstream of CIITA in this regulatory pathway. The human and murine forms of the IK cytokine share a sequence identity of 98% at the amino-acid level. The murine IK cytokine consists of 557 amino-acid residues and contains a trimeric coiled-coil motif, a nuclear localization signal, and a region rich in tandem repeats of arginine (R)-aspartic acid (D) or R-glutamic acid (E), which are commonly found in RNA-binding nuclear proteins [8]. These findings imply that the IK cytokine acts as a regulatory protein and is possibly involved in CIITA transcription.

In a previous study, truncated IK (tIK), produced by the deletion of 315 amino-acid residues from the N-terminal end of IK, was reported to be incapable of localizing to the nucleolus, although full-length IK exhibits efficient sorting and nucleolar localization [8]. Thus, IK acts not only as a nuclear binding protein, but also as a secretory protein when translated from methionine at position 316. In a study of lupus nephritis (kidney inflammation), both tIK and full-length IK reduced interferon-γ-induced MHC class II expression to a similar extent, and tIK-treated mice experienced less kidney damage [9]. Transgenic mice expressing tIK were also resistant to the induction of inflammatory arthritis, due to a reduction in pro-inflammatory immune cells such as macrophages and pathogenic T helper 17 (Th17) cells [10, 11]. In addition, treating cultured T cells with tIK protein induced the expression of A20, a negative regulator of nuclear factor-κB (NF-κB)-induced inflammation, and reduced the expression of several transcription factors related to T-cell activation. In particular, A20 restricts NF-κB activation via a negative feedback loop through its deubiquitinase function, by targeting various signaling proteins dependent on NF-κB stimulation [12]. Therefore, A20 is considered an attractive therapeutic target for inflammatory and autoimmune diseases. These findings suggest that exogenous IK and/or tIK may be a candidate treatment for inflammatory arthritis, including rheumatoid arthritis (RA).

In the present study, given that tIK suppresses inflammatory arthritis by inhibiting the expression of the MHC gene, we constructed monomeric and dimeric structural models of the human tIK protein via computational modeling. The molecular structure of a putative anti-inflammatory peptide derived from human tIK was designed such that the peptide had a significant inhibitory effect on Th17 cell differentiation and tumor necrosis factor-α (TNF-α) secretion compared to the control [vasoactive intestinal peptide (VIP) or dexamethasone (DMS)]. We further employed circular dichroism (CD) to analyze in silico structures, to predict the structural properties of the peptide. In addition, we measured the hemolytic activity of the peptide to assess its toxicity and verify its safety in mouse erythrocytes. To summarize, we investigated the inhibitory effects of a human tIK-derived peptide on two pro-inflammatory cytokines, interleukin (IL)-17 and TNF-α, and evaluated the feasibility of using this peptide in skincare products.

## Materials and Methods

### Protein Structure Modeling

Protein structure modeling took place over four stages: fold assignment, target-template alignment, comparative model building, and model evaluation. First, we attempted to find all known relevant protein structures in the Protein Data Bank (PDB), for as many domains in the modeled sequence as possible (fold recognition or fold assignment). The domain folds in the target sequence can be assigned using pairwise and multiple sequence similarity searches, as well as by using methods that rely explicitly on the known structures of the candidate template proteins. Although fold assignment predicts a structural relationship between two proteins, it does not produce an explicit three-dimensional (3D) model of the target sequence. Therefore, fold assignment is generally followed by alignment of the target sequence with one or more template structures, to establish the best possible correspondence between residues in the target and template sequences.

After alignment, the next step is comparative model building, to produce explicit 3D models of the aligned domains of the target protein based on the alignment with the template structures. In these models, the main and side chains usually consist entirely of non-hydrogen atoms, including insertions and deletions relative to the template structures. Finally, the models are evaluated by considering structural and energetic criteria, rather than sequence similarity alone. Model evaluation helps in the assessment of the types of information that can be extracted from each model. If all models are unsatisfactory, the cycle of fold assignment, alignment, modeling, and model evaluation is repeated until a satisfactory model is produced. A useful approach to fold assignment and alignment is to accept uncertain instances of both, build a full-atom comparative model of the target sequence, and then decide whether the match and alignment are accurate by evaluating the resulting comparative model.

In the case of the human tIK protein, which is a cytokine, no tertiary structure has been reported to date. Therefore, we conducted a 3D structural study based on the protein-structure modeling steps described above. First, we conducted a fold-assignment search, using PSI-BLAST, for proteins that are homologous with tIK and used them as template proteins for homology modeling. From the PSI-BLAST search, a number of proteins homologous with tIK were selected as template proteins, because no protein was homologous with tIK over its entire region. Then, protein-structure modeling was conducted using multiple sequence alignment (MSA). The sequence alignment produced by MSA and the 3D structures of the template proteins were used as input data in homology modeling, and a putative 3D structural model of tIK was produced after a refinement process.

### Peptide Preparation

The peptides used in this study were synthesized by AnyGen Co., Ltd. (Korea) using the standard Fmoc solid-phase peptide synthesis protocol and commercially available amino acids. Peptides were purified via reverse-phase high-performance liquid chromatography (LabSolutions; Shimadzu, Japan) using a C18 analytical column. Elution was carried out with a water-acetonitrile linear gradient [5-65% (v/v) of acetonitrile] containing 0.05% (v/v) trifluoroacetic acid (Sigma-Aldrich, USA). The molecular weight of the purified peptide was confirmed using matrix-assisted laser desorption/ionization time-of-flight mass spectrometry (AXIMA-Assurance; Shimadzu). The concentration of dissolved peptide was determined using a BCA protein assay kit (Thermo Scientific, USA).

### Th17 Cell Differentiation

Naïve CD4^+^ T cells were isolated from the spleen of wild-type Balb/c mice via magnetic-activated cell sorting using a CD4^+^ T Cell Isolation Kit (Miltenyi Biotec, Germany), in accordance with the manufacturer’s instructions. The RPMI medium for stimulating Th17 cell differentiation included 10 μg/ml anti-CD3 (eBioscience, USA), 1.5 μg/ml anti-CD28 (eBioscience), 5 ng/ml transforming growth factor-β (R&D Systems, USA), and 20 ng/ml IL-6 (BioLegend, USA). After cells had been cultured in this conditioned medium for 2 days at 37°C, 100 μl of the supernatant was analyzed for mouse IL-17 (mIL-17) using an IL-17-specific enzyme-linked immunosorbent assay (ELISA). All tests were repeated three times independently, and the means and standard deviations (SDs) from three tests were calculated.

### TNF-α Assays

The murine monocyte/macrophage cell line J774A.1 was obtained from the American Type Culture Collection (ATCC, USA), grown in Dulbecco’s modified Eagle’s medium (Gibco Laboratories, USA), and cultured at 37°C in humidified 5% CO_2_. The culture medium was supplemented with 10% fetal bovine serum and 2 mM glutamine. The cells were seeded into 24-well culture plates at a density of 1 × 10^5^/ml and allowed to adhere overnight. Thereafter, the medium was replaced with fresh medium. The cells were pre-treated with DMS or a peptide (P1, P2, P3, P4, or P4S) and then stimulated with 100 ng/ml of lipopolysaccharide (LPS). Supernatants were collected after 24 h and stored at -20°C. Concentrations of mouse TNF-α (mTNF-α) were measured using ELISA (Mouse TNF-α DuoSet ELISA Kit; R&D Systems Europe, UK) according to the manufacturer’s instructions. All tests were repeated three times independently, and the results are expressed as means ± SDs.

### Peptide Structural Characterization

The secondary structures of P4 and its analogue were analyzed via CD spectroscopy, which was performed using a Jasco J-815 spectropolarimeter (Japan) with the peptides dissolved in deionized water in a quartz cell with a path length of 1 mm. Wavelengths between 190 and 250 nm were measured at a bandwidth of 1 nm with a step resolution of 0.5 nm, sensitivity of 20 mdeg, and speed of 50 nm/min. Each spectrum was obtained as an average of five scans with a response time of 0.25 s at ambient temperature, and the baseline was corrected by subtracting the value obtained for deionized water without peptides. Three-dimensional models of P4 and P4S were also constructed via in silico prediction using the online tool PEP-FOLD3 (http://bioserv.rpbs.univ-paris-diderot.fr/services/PEP-FOLD3) [13]. PEP-FOLD3 has an improved and fast de novo peptide structural modeling engine, such that a wide range of peptide sizes (5–50 amino acids) can be considered. The prediction process consists of three main steps. Starting from an amino-acid sequence, the first step is to predict the prior probabilities ([SA] sequence alignment profile) of each peptide fragment associated with each of 27 SA states. In the second step, one conformation is generated from each series of states using a rigid assembly procedure and prototype fragments. In the third step, clusters are identified, and the conformations are scored.

### Hemolytic Activity Assays

Hemolytic activity was assayed as previously described [14]. Mouse erythrocytes (Innovative Research, Inc., USA) were rinsed three times with phosphate-buffered saline (PBS) and then suspended in PBS to a concentration of 8% (v/v). The erythrocyte suspension was seeded into 96-well culture plates and incubated with various concentrations of peptides for 1 h at 37°C. The erythrocyte suspension was then centrifuged at 270 ×*g* for 15 min, and the supernatant was collected after another 1 h of incubation. The absorbance at 414 nm was recorded using an ELISA reader. For the assays, 0.1% (v/v) Triton X-100 was used as the 100% hemolytic activity control. The erythrocyte hemolysis rate was calculated as follows [15]:

Hemolysis rate (%) = {(Sample absorbance − PBS absorbance)/(0.1% Triton X-100 absorbance − PBS absorbance)} × 100

Hemoglobin release after peptide treatment was assessed by measuring the sample absorbance. Positive and negative controls representing total or no hemolysis were established by treating mouse erythrocytes with 0.1% (v/v) Triton X-100 and PBS, respectively.

### Statistical Analysis

All data are presented as the mean ± SD for each treatment group in a given experiment. All experiments were performed at least three times independently. Treatment groups were compared to an appropriate control group, and the statistical significance of differences between groups was evaluated using a two-tailed paired t-test. Differences were considered significant at *p* < 0.05.

## Results

### Designing Peptides from the tIK Protein

Similar to full-length IK [9], the soluble tIK (M316-Y557) protein inhibits MHC class II expression and reduces the abundance of pro-inflammatory immune cells (*e.g.*, Th17) [10, 11]. Based on these previous results, we decided to model the tIK structure, but not that of full-length IK. From the computational modeling process, a monomeric structural model of the human tIK protein composed of 242 amino acids was produced. To derive putative anti-inflammatory peptides from the human tIK protein, we subsequently constructed a dimeric tIK model based on the structure of human IL-10 (hIL-10), which is a typical anti-inflammatory cytokine (see Fig. S5 of Choi *et al*. [11]). A monomer of IL-10 (PDB ID: 1Y6K) from *Homo sapiens* was selected as the search model because, among the immunosuppressive cytokines that show promise for treating inflammatory diseases, IL-10 is secreted by many cell populations and is thus a highly representative cytokine. IL-10 plays an important role in the regulation of inflammatory responses, as well as in the differentiation and proliferation of immune cells [16, 17]. In particular, levels of IL-10 increased in bone-derived macrophages isolated from tIK-transgenic mice after LPS treatment compared to those isolated from wild-type mice [10], indicating that the tIK protein plays at least some role in protecting against the inflammation caused by macrophage activation. Therefore, we expected that tIK would be closely associated with IL-10, *i.e.*, that tIK and IL-10 structures would be similar, and that tIK and the IL-10 receptor (IL-10R) would also share a close structural relationship.

**Fig. 2:**
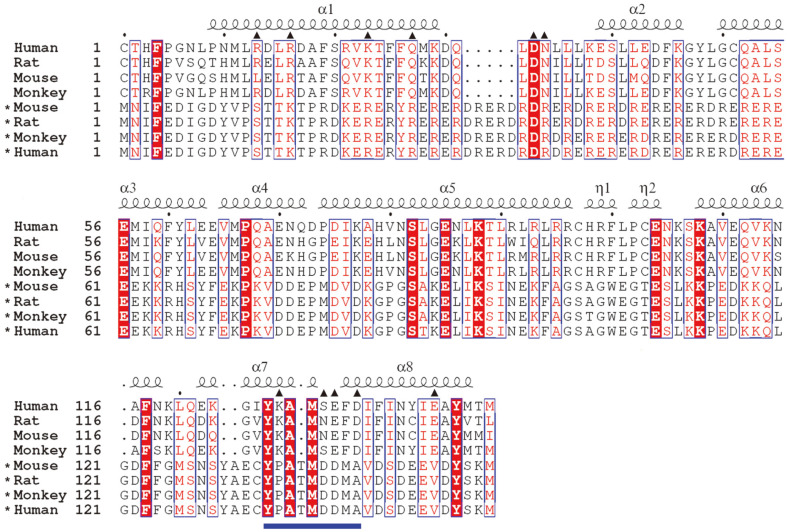
Sequence alignment of truncated inhibitor K562 (tIK) proteins and interleukin (IL)-10 from different species. The amino-acid sequences of IL-10 and tIK were compared pairwise. The sequences used in this alignment are those of human (GenBank accession no. NM_000572.3), rat (NP_036986.2), mouse (NP_034678.1), and monkey (NP_001038192.1) IL-10, and *mouse (NP_036009.1), *rat (NP_001005537.1), *monkey (NP_001243826.1), and *human (NP_006074.2) tIK. Highly conserved residues are indicated by red text and boxed in blue, whereas strictly conserved residues are shown against a red background. Residues that interact with the IL-10 receptor (IL-10R) at the active site are indicated by filled triangles. The secondary-structure elements found in complexed hIL-10 are shown for each corresponding sequence. The P4S peptide (YPATMDDMA) designed in this study is indicated with a blue bar. The figure was prepared using ESPript.

Human tIK and hIL-10 had a similar V-shape configuration and exhibited a high sequence homology of 47.6%. In addition, IL-10 and tIK sequences did not vary noticeably among different species (Fig. 2). Examination of the hIL-10 residues that interact with IL-10R at the active site revealed that most of the residues are found at the N- and C-terminal regions of hIL-10. We also predicted that human tIK residues would interact with IL-10R at a location structurally similar to that used by hIL-10, based on the putative structural relationship between human tIK and IL-10R. Hence, its motif sites were selected as candidates for peptide fragments derived from human tIK. Four candidate peptides were determined via structure-based sequence alignment with tIK, based on the interacting residues in the hIL-10/IL-10R complex. The sequences of the candidate peptides were as follows: P1, TKTPRDKERERYRERERDRERDRDRDRERER; P2, GMSNSYAECYPATMDDMAVDSDEEVDYSKMD; P3, TPRDKERERYRERERDRERDRDRDRER; and P4, YPATMDDMAVDSDEEVDY. P3 and P4 are peptide fragments of P1 and P2, respectively.

### Inhibitory Effects on Th17 Cell Differentiation

**Fig. 3:**
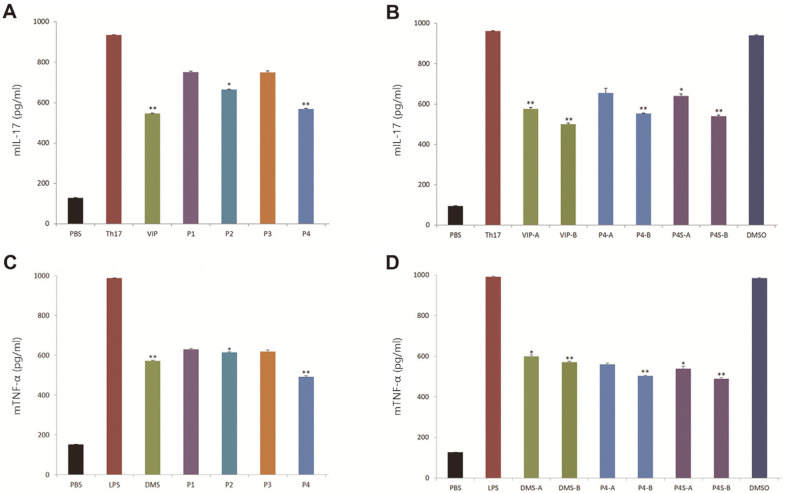
Inhibition of differentiation into T helper 17 (Th17) cells and mouse tumor necrosis factor-α (mTNF-α) secretion by J774A.1 cells induced by candidate peptides. Concentrations of mouse interleukin-17 (mIL-17) and mTNF-α in cells exposed to four candidate peptides (P1–P4) and one analogue peptide (P4S) derived from the human tIK protein were determined using enzyme-linked immunosorbent assay. (**A, C**) Treatment with 1 μg/ml of P1, P2, P3, P4, vasoactive intestinal peptide (VIP), or dexamethasone (DMS). (**B, D**) Treatment with 0.1 μg/ml of VIP-A, P4-A, P4S-A, or DMS-A; or 1 μg/ml of VIP-B, P4-B, P4S-B, or DMS-B, using 0.1% dimethyl sulfoxide (DMSO). The A and B forms of VIP, P4, and P4S are identical peptides with the same sequences. All experiments were independently repeated three times. Data are presented as means ± standard deviations (*n* = 3).

Four peptides were derived based on the human tIK model structure, and their anti-inflammatory effects were analyzed using the Th17 cell differentiation model. Treatment with candidate peptides suppressed cell differentiation to Th17 cells relative to the control group, in which Th17 cell differentiation was induced by a differentiation agent. Additionally, we compared peptide anti-inflammatory activity with that of commercially available VIP, which was used as a positive control. VIP is a potent anti-inflammatory agent that regulates intestinal epithelial barrier homeostasis [18]. Of the four peptides, P4, a peptide fragment of P2, exhibited an inhibition rate of 39.1%, similar to that of VIP (41.4%), whereas P2, which is composed of 31 amino acids, exhibited an inhibition rate of 28.8%. P1 and P3 exhibited similarly low inhibition rates of 19.6% and 19.7%, respectively (Fig. 3A).

**Fig. 4:**
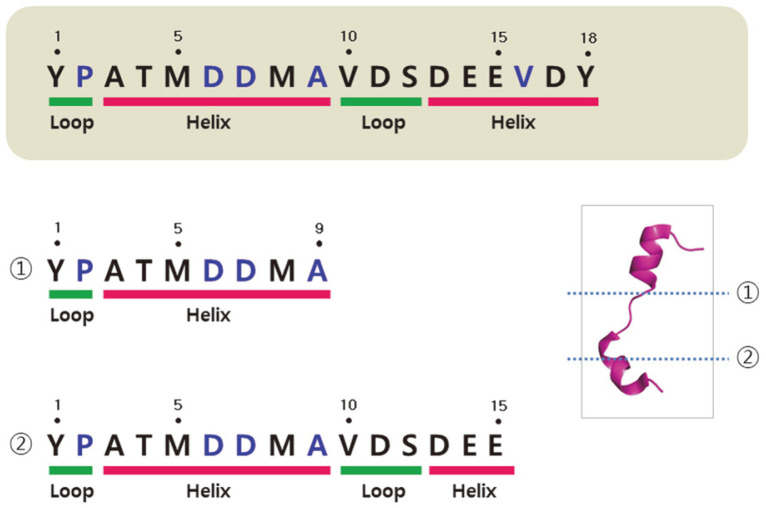
In silico prediction of peptide secondary structure using the PEP-FOLD3 tool. The P4 (YPATMDDMAVDSDEEVDY) peptide was predicted to have a helix-loop-helix form, and P4S (YPATMDDMA) was predicted to consist of the first nine residues from the P4 N terminus, and to have a loop-helix configuration as its secondary structure. Blue letters indicate the human tIK residues that interact with the interleukin-10 receptor (IL-10R) at a location structurally similar to that used by human interleukin-10 (hIL-10), based on the putative structural relationship between human tIK and IL-10R. The figure was prepared using PyMOL.

Subsequently, P4 was selected as the primary candidate peptide, as it only consists of 18 amino acids and is thus easier and cheaper to produce via fragmentation. Secondary-structure analysis and sequence alignment revealed that P4 has a helix-loop-helix structure and interestingly, the region from the N terminus to the ninth amino acid includes most of the key residues (including the α7 C-terminal region, α7/α8 loop, and α8 N-terminal region) involved in binding hIL-10 to IL-10R (Figs. 2 and 4). Hereafter, we refer to this human tIK-derived peptide as P4S, as it is a fragment homolog of P4. We performed a second anti-inflammatory experiment using VIP, P4, and P4S (YPATMDDMA), and found that treatment with 1.0 μg/ml of P4S (43.8%) or P4 (42.4%) strongly inhibited mIL-17 (Fig. 3B). The control group received 0.1% dimethyl sulfoxide, which did not adversely affect the Th17 cells.

### Quantitative Analysis of mTNF-α Using ELISA

The anti-inflammatory properties of the candidate peptides were also assessed in vitro by measuring mTNF-α concentrations using ELISA. TNF-α is a cell-signaling protein and pro-inflammatory cytokine involved in systemic inflammation; it is among the cytokines that participate in acute-phase reactions. DMS, which was used as a positive control in our in vitro tests, is employed to treat many inflammatory and autoimmune conditions, such as RA and bronchospasm. Initially, J774A.1 cells were exposed to one of four candidate peptides, and the cells were subsequently stimulated with LPS. Across all peptides, mTNF-α secretion was inhibited by more than 36%compared to the LPS-only group (Fig. 3C). In particular, the P4 peptide exhibited an inhibition rate of 50.2%against mTNF-α, higher than those of the other three peptides (Fig. 3C). Treatment with 0.1 and 1.0 μg/ml of P4S, a peptide fragment of P4, strongly inhibited mTNF-α, by 45.5% and 50.7%, respectively (Fig. 3D). Further structural and mechanistic studies are needed to understand how the P4 and P4S peptides inhibit mTNF-α.

### Peptide Secondary Structure

**Fig. 5:**
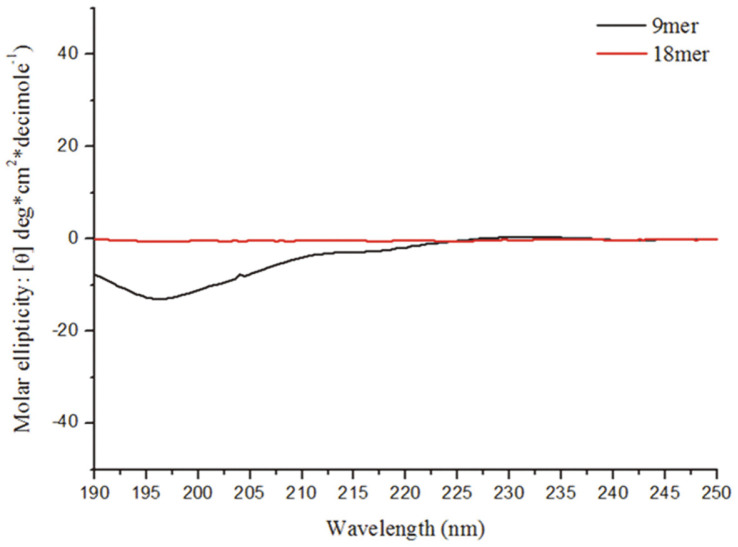
Circular dichroism spectra of two target peptides, P4 (18-mer; red) and P4S (9-mer; black). All experiments were independently repeated three times.

In silico predictions were made using the PEP-FOLD3 online tool (https://bioserv.rpbs.univ-paris-diderot.fr/services/PEP-FOLD3/). The predicted 3D model of P4 indicated that its structure consisted of a helix-loop-helix with 18 residues. P4S was predicted to have a loop-helix configuration as its secondary structure, with seven residues (3-Ala to 9-Ala) appearing to make up a short helix structure (Fig. 4). Generally, secondary structures are observed in a localized part of a protein. Considering the longer length of the P4 peptide, the short helical region predicted for P4S might have been the product of division due to the formation of an irregular secondary structure. In particular, the residues alanine and methionine tend to form helices in secondary structures. We extracted the P4S fragment (YPATMDDMA) from the P4 peptide sequence based on its predicted secondary structure (Fig. 4), and tested it as a candidate peptide due to its homology with P4. To examine the secondary structures and behaviors of P4 and P4S in an aqueous environment, the CD spectra of both peptides were measured. In aqueous solution, P4 is an unstructured peptide and P4S has a random coil structure with a minimum at approximately 196 nm (Fig. 5). Thus, the P4S peptide, which exerts inhibitory effects on mIL-17 and mTNF-α, has a random coil configuration.

### Peptide Hemolytic Activity

**Fig. 6:**
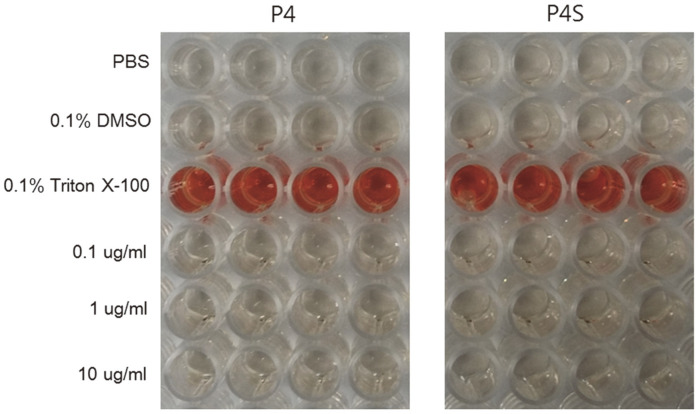
Hemolytic activities of the P4 and P4S peptides. The absorbance of each cell was measured at 414 nm (Abs 414 nm) and the hemolysis rate (%) was calculated using the following equation: hemolysis rate (%) = {(Abs 414 nm for each peptide treatment - Abs 414 nm for PBS) / (Abs 414 nm for 0.1% Triton X-100 treatment - Abs 414 nm for PBS)} × 100. As a positive control, 0.1% of Triton X-100 was used to represent 100% hemolysis. PBS, phosphate-buffered saline.

Peptide toxicity was evaluated by measuring mouse erythrocyte lysis and hemoglobin release upon exposure to the P4 and P4S peptides (Fig. 6). The hemolytic activities of P4 and P4S against mouse erythrocytes were quantified using a colorimetric assay. No hemolytic activity against mouse erythrocytes was observed at any dose of P4 or P4S (0.1, 1, and 10 μg/ml).

## Discussion

In this study, we designed a peptide from human tIK protein that can inhibit the differentiation of pathogenic Th17 cells, which are involved in the development of RA. Th17 cells and the IL-17 signaling pathway are key for the induction of autoimmune diseases; hence, they are common therapeutic targets [19-22]. Recently, the ability of a novel 23 amino-acid anti-inflammatory peptide (KS23) derived from adiponectin to mitigate experimental autoimmune uveitis (EAU) was evaluated. KS23 significantly improved EAU-associated histopathological scores and decreased the expression of pro-inflammatory cytokines (interferon [IFN]-γ, TNF-α, IL-6, and IL-17A), chemokines (LARC, RANTES, MIG, and IP-10), and chemokine receptors (CCR6 and CXCR3). Additionally, Th1 and Th17 cells were suppressed following intraperitoneal injections of KS23, suggesting that this peptide is a promising therapeutic agent [23].

**Fig. 7:**
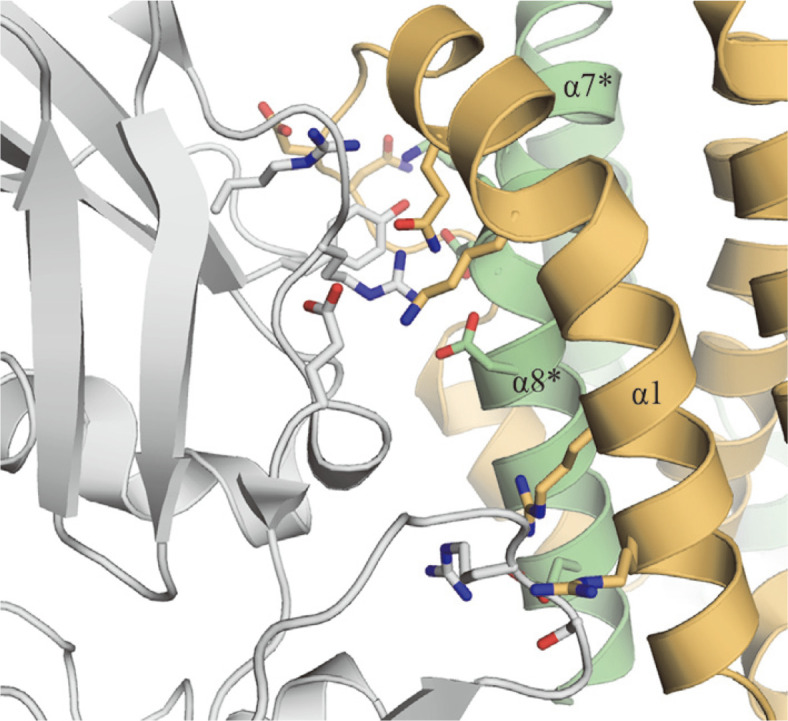
Structure of the binary complex formed by human interleukin-10 (hIL-10) and the interleukin-10 receptor (IL-10R). The active site in the hIL-10/IL-10R complex (PDB ID: 1Y6K) is magnified for greater clarity. In the crystal, hIL-10 exists as a dimer positioned on a crystallographic two-fold axis. Dimeric hIL-10 is shown in each subunit in a different color. Key residues in the hIL-10/IL-10R complex are represented by colored stick models. The residues in the N-terminal region (α1) in subunit A and the C-terminal region (α7* and α8*) in subunit B interact with the IL-10R residues (gray).

IL-17 is a cytokine produced by Th17 cells differentiated from CD4^+^ cells and plays a key role in inducing inflammation. We decided to develop an anti-inflammatory peptide derived from human IK protein, as peptides are relatively easier and cheaper to manufacture than proteins. A dimeric tIK protein model was constructed based on the hIL-10 structure. IL-10 is a typical anti-inflammatory cytokine that inhibits the activity of various immune cells including Th1 cells, macrophages, and natural killer (NK) cells, and also suppresses the expression of inflammation-associated genes [24, 25]. In addition, IL-10, similar to IK, downregulates the expression of MHC class II in monocytes and reduces antigen-specific T-cell proliferation [26]. Therefore, IL-10 is a key regulator of immune system homeostasis and has been investigated as a candidate modulator of various chronic inflammatory diseases [27]. Based on this information, we predict that IK is closely associated with IL-10 and, based on its structure, may be part of an IL-10 subfamily. Subsequently, the region containing the hIL-10 active-site residues that interact with its receptor was examined via sequence alignment with the human tIK sequence (Figs. 2 and 7).

The IL-17 family of cytokines are strong inducers of inflammation and help protect the body against invading pathogens. However, they also produce adverse side effects and contribute to the tissue destruction that characterizes chronic inflammatory and autoimmune diseases, such as psoriasis, RA, and multiple sclerosis. In the present study, P4S, a peptide fragment of P4, inhibited mIL-17 to a similar degree as VIP, a potent anti-inflammatory agent (Fig. 3B). Because P4S is smaller than P4 (9 vs. 18 amino acids), it can be produced industrially through organic synthesis and is suitable for large-scale applications.

Moreover, P4S treatment significantly inhibited the secretion of mTNF-α, a pro-inflammatory cytokine, compared to LPS treatment (Fig. 3D). According to Kelhälä *et al*. [28], Th17 cells and TNF-α expression are linked, with increased expression of TNF-α shown to be important in Th17 cell activation. Furthermore, TNF-α can act synergistically with IL-17 to promote inflammation in psoriasis, while IL-17 and IFN-γ can act synergistically to produce pro-inflammatory cytokines in keratinocytes [29, 30]. In this study, treatment with P4S reduced mIL-17 and mTNF-α secretion. The results indicate that, when activated, the P4S peptide can play a vital role in disease processes associated with inflammation. Our findings also provide clues regarding new therapeutic targets for inflammatory activation. From a safety perspective, we found that P4 and P4S did not exhibit hemolytic activity at concentrations below 10 μg/ml (Fig. 6). As such, P4 or P4S may be biologically safe and suitable for anti-inflammatory treatment, and could also reduce the risk of inflammation due to hemolysis, which is involved in several diseases [31].

In conclusion, the results from in vitro tests indicated that the human tIK protein-derived P4S peptide exerts inhibitory effects against two pro-inflammatory cytokines, mIL-17 and mTNF-α. This novel putative anti-inflammatory peptide also exhibits low toxicity to mouse erythrocytes. Therefore, P4S may be a useful raw material for the cosmetics industry, specifically for beauty and skincare products. Based on its properties, P4S could indirectly participate in repairing damaged skin and promoting wound healing; hence, the novel peptide may also be beneficial for treating skin diseases if incorporated into cosmetic products.

## References

[1] Holling TM, Schooten E, Den Elsen PJ (2004). Function and regulation of MHC class II molecules in T-lymphocytes: of mice and men. Hum. Immunol..

[2] Neefjes J, Jongsma ML, Paul P, Bakke O (2011). Towards a systems understanding of MHC class I and MHC class II antigen presentation. Nat. Rev. Immunol..

[3] Gaspari AA, Jenkins MK, Katz SI (1988). Class II MHC-bearing keratinocytes induce antigen-specific unresponsiveness in hapten-specific Th1 clones. J. Immunol..

[4] Frasca L, Marelli-Berg F, Imami N, Potolicchio I, Carmichael P, Lombardi G (1998). Interferon-gamma-treated renal tubular epithelial cells induce allospecific tolerance. Kidney Int..

[5] Bal V, Mclndoe A, Denton G, Hudson D, Lombardi G, Lamb J (1990). Antigen presentation by keratinocytes induces tolerance in human T cells. Eur. J. Immunol..

[6] Cella M, Engering A, Pinet V, Pieters J, Lanzavecchia A (1997). Inflammatory stimuli induce accumulation of MHC class II complexes on dendritic cells. Nature.

[7] Vedrenne J, Assier E, Pereno R, Bouzinba-Segard H, Azzarone B, Jasmin C (1997). Inhibitor (IK) of IFN-γ induced HLA class II antigens expression also inhibits HLA class II constitutive expression in the human Raji B cell line. Oncogene.

[8] Assier E, Bouzinba-Segard H, Stolzenberg MC, Stephens R, Bardos J, Freemont P (1999). Isolation, sequencing and expression of RED, a novel human gene encoding an acidic-basic dipeptide repeat. Gene.

[9] Muraoka M, Hasegawa H, Kohno M, Inoue A, Miyazaki T, Terada M (2006). IK cytokine ameliorates the progression of lupus nephritis in MRL/*lpr* mice. Arthritis Rheum..

[10] Park HL, Lee SM, Min JK, Moon SJ, Kim I, Kang KW (2017). IK acts as an immunoregulator of inflammatory arthritis by suppressing T_H_17 cell differentiation and macrophage activation. Sci. Rep..

[11] Choi S, Park HL, Jung S, Kim EK, Cho ML, Min JK (2017). Therapeutic effect of exogenous truncated IK protein in inflammatory arthritis. Int. J. Mol. Sci..

[12] Shembade N, Harhaj EW (2012). Regulation of NF-κB signaling by the A20 deubiquitinase. Cell Mol. Immunol..

[13] Lamiable A, Thévenet P, Rey J, Vavrusa M, Derreumaux P, Tufféry P (2016). PEP-FOLD3: faster de novo structure prediction for linear peptides in solution and in complex. Nucleic Acids Res..

[14] Evans BC, Nelson CE, Yu SS, Beavers KR, Kim AJ, Li H (2013). Ex vivo red blood cell hemolysis assay for the evaluation of pH-responsive endosomolytic agents for cytosolic delivery of biomacromolecular drugs. J. Vis. Exp..

[15] Molly FC, Sean WD, Mark SW, Colleen MS, Scott AS, Don RP (2000). Standard practice for assessment of hemolytic properties of materials. American Society for Testing of Materials. ASTM F756-00.

[16] Asadullah K, Sterry W, Volk HD (2003). Interleukin-10-review of a new approach. Pharmacol. Rev..

[17] Ng TH, Britton GJ, Hill EV, Verhagen J, Burton BR, Wraith DC (2013). Regulation of adaptive immunity; the role of interleukin-10. Front. Immunol..

[18] Seo S, Miyake H, Alganabi M, Janssen Lok M, O'Connell JS, Lee C (2019). Vasoactive intestinal peptide decreases inflammation and tight junction disruption in experimental necrotizing enterocolitis. J. Pediatr. Surg..

[19] Gaffen SL (2009). Role of IL-17 in the pathogenesis of *rheumatoid arthritis*. Curr. Rheumatol. Rep..

[20] Jin W, Dong C (2013). IL-17 cytokines in immunity and inflammation. Emerg. Microbes Infect..

[21] Shabgah AG, Fattahi E, Shahneh FZ (2014). Interleukin-17 human inflammatory diseases. Postepy Dermatol. Alergol..

[22] Paradowska-Gorycka A, Haladyj E (2015). Th17-cells in the pathogenesis of rheumatoid arthritis. Int. J. Autoimmune Disord. Ther..

[23] Niu T, Cheng L, Wang H, Zhu S, Yang X, Liu K (2019). KS23, a novel peptide derived from adiponectin, inhibits retinal inflammation and downregulates the proportions of Th1 and Th17 cells during experimental autoimmune uveitis. J. Neuroinflammation.

[24] Murray PJ (2005). The primary mechanism of the IL-10-regulated anti-inflammatory response is to selectively inhibit transcription. Proc. Natl. Acad. Sci. USA.

[25] Couper KN, Blount DG, Riley EM (2009). IL-10: The master regulator of immunity to infection. J. Immunol..

[26] Koppelman B, Neefjes JJ, de Vries JE, Malefyt RW (1997). Interleukin-10 down-regulates MHC class II αβ peptide complexes at the plasma membrane of monocytes by affecting arrival and recycling. Immunity.

[27] Sanjabi S, Zenewicz LA, Kamanaka M, Flavell RA (2010). Anti- and pro-inflammatory roles of TGF-β, IL-10, and IL-22 in immunity and autoimmunity. Curr. Opin. Pharmacol..

[28] Kelhälä HL, Palatsi R, Fyhrquist N, Lehtimäki S, Väyrynen JP, Kallioinen M (2014). IL-17/Th17 pathway is activated in acne lesions. PLoS One.

[29] Chiricozzi A, Guttman-Yassky E, Suárez-Farinas M, Nograles KE, Tian S, Cardinale I (2011). Integrative responses to IL-17 and TNF-α in human keratinocytes account for key inflammatory pathogenic circuits in psoriasis. J. Invest. Dermatol..

[30] Teunissen MB, Koomen CW, de Waal Malefyt R, Wierenga EA, Bos JD (1998). Interleukin-17 and interferon-gamma synergize in the enhancement of proinflammatory cytokine production by human keratinocytes. J. Invest. Dermatol..

[31] Hilary S, Habib H, Souka U, Ibrahim W, Platat C (2017). Bioactivity of arid region honey: an in vitro study. BMC Complement Altern. Med..

